# A restatement of the natural science evidence base regarding the source, spread and control of *Campylobacter* species causing human disease

**DOI:** 10.1098/rspb.2022.0400

**Published:** 2022-06-29

**Authors:** Matthew R. Goddard, Sarah O'Brien, Nicola Williams, Javier Guitian, Andrew Grant, Alison Cody, Frances Colles, Jean-Charles Buffet, Ella Adlen, Andrea Stephens, H. Charles J. Godfray, Martin C. J. Maiden

**Affiliations:** ^1^ School of Life Sciences, Joseph Banks Laboratories, University of Lincoln, Lincoln LN6 7DL, UK; ^2^ School of Natural and Environmental Sciences, Ground floor, Agriculture Building, Newcastle University, Newcastle upon Tyne NE1 7RU, UK; ^3^ Institute of Infection, Veterinary and Ecological Sciences, University of Liverpool, Leahurst Campus, Neston, Wirral CH64 7TE, UK; ^4^ Veterinary Epidemiology, Economics and Public Health, Royal Veterinary College, Hawkshead Lane, North Mymms, Hatfield AL9 7TA, UK; ^5^ Department of Veterinary Medicine, University of Cambridge, Madingley Road, Cambridge CB3 0ES, UK; ^6^ Department of Zoology, University of Oxford, 11a Mansfield Road, Oxford OX1 3SZ, UK; ^7^ Oxford Martin School, University of Oxford, 34 Broad Street, Oxford OX1 3BD, UK

**Keywords:** *Campylobacter*, campylobacteriosis, food safety, poultry, epidemiology

## Abstract

Food poisoning caused by *Campylobacter* (campylobacteriosis) is the most prevalent bacterial disease associated with the consumption of poultry, beef, lamb and pork meat and unpasteurized dairy products. A variety of livestock industry, food chain and public health interventions have been implemented or proposed to reduce disease prevalence, some of which entail costs for producers and retailers. This paper describes a project that set out to summarize the natural science evidence base relevant to campylobacteriosis control in as policy-neutral terms as possible. A series of evidence statements are listed and categorized according to the nature of the underlying information. The evidence summary forms the appendix to this paper and an annotated bibliography is provided in the electronic supplementary material.

## 1. Introduction

The consumption of food and drink contaminated with *Campylobacter* bacteria can cause campylobacteriosis in humans. While food may be made safe with adequate cooking, and by avoiding cross-contamination during food preparation, *Campylobacter* is the most common cause of acute bacterial gastroenteritis both in the UK and globally [1]. Campylobacteriosis is chiefly a sporadic disease with many isolated cases that usually peak in early summer in the UK, though there are occasional larger outbreaks [2]. Most people who become infected with *Campylobacter* suffer from illness and discomfort and require time to convalesce, but severe disease and death can occur. The use of antibiotics is only recommended for those at greatest risk of severe disease or death from campylobacteriosis (chiefly the young, old and immune compromised). In other patients, antibiotics only shorten the disease by a few days and their prescription may accelerate the evolution of antibiotic resistance, which has already been observed in *Campylobacter*. The total cost to society of foodborne campylobacteriosis is estimated at over £700 million per annum in the UK alone [3].

A suite of producer, food-chain and public health measures have been implemented to attempt to reduce the levels of campylobacteriosis in the UK, particularly targeting poultry, which has been identified as the main source of human infection. Surveys indicate that levels of *Campylobacter* in fresh poultry at retail outlets in the UK have decreased in recent years, but reported human *Campylobacter* infections have remained relatively constant [4]. Further interventions are needed to limit the individual and economic impacts of campylobacteriosis, though each imposes different levels of costs on livestock production, processing and retail sectors. Designing better control measures without unnecessary costs requires a better understanding of the origin and transmission dynamics of the *Campylobacter* species causing human disease.

The aim of this ‘Restatement' is to present a clear and succinct summary of the evidence for the source and spread of *Campylobacter* in the food chain and how it might be controlled. We focus on the UK although the evidence base is relevant to many other countries, particularly those in temperate regions. The Restatement is written for an informed but not expert audience, for example, senior policy-makers with food safety in their brief. We also highlight areas where the evidence base is poorly developed to assist policy makers. In a policy area that can be contentious, we aim to be as policy-neutral as possible in the compilation and presentation of evidence.

## 2. Material and methods

The relevant literature on *Campylobacter* was reviewed with particular focus on studies in the UK and a first draft evidence summary was produced by a subset of the authors. At a workshop, all authors met to discuss the different evidence statements and to assign a description of the nature of the evidence to each statement using a restricted set of terms. The statements and their assessments were subsequently debated via correspondence until a consensus was achieved. We use the following restricted terms to describe the evidence, indicated by abbreviated codes, which are similar to those used in previous Restatements.

**[S_trong_]:** A **strong** evidence base likely involving multiple experimental studies or field data collections, with appropriate detailed statistical or other quantitative analysis.

**[L_imited_]: Limited evidence** from perhaps only one or few studies, with further studies needed to strengthen the evidence base.

**[E_xp_O_p_]:** A consensus of **expert opinion** extrapolating results from related systems and well-established epidemiological and pathological principles.

**[P_roj_]**: **Projections** based on the available evidence for which substantial uncertainty often exists.

## 3. Results

The summary of the natural science evidence base relevant to *Campylobacter* control policy-making in the UK is given in the appendix, with an extensive annotated bibliography provided as electronic supplementary material.

## 4. Discussion

The most important source of *Campylobacter* that cause human disease is meat from farmed animals such as cattle, pigs and particularly broiler chickens. *Campylobacter* infect the intestines of most farmed animals and are regularly found on fresh carcasses, particularly the carcasses of broiler chickens, and it is likely that bacteria in digestive tracts are spread to carcasses during slaughter and factory processing. Live bacteria on meat and carcasses may be ingested by humans via cross-contamination to other foods and items if food preparation hygiene is poor prior to cooking, or if meat is not cooked sufficiently.

Poultry is the most consumed meat in the UK and a major source of *Campylobacter*. *Campylobacter* from non-poultry livestock, particularly ruminants, are also a significant cause of human disease, as is increasingly shown by genetic source-attribution studies including recent studies using whole-genome sequencing [5]. The evidence base for *Campylobacter* levels on retail beef, lamb and pork, and the effect of food-chain interventions designed to reduce these, is less well developed than for poultry. The Restatement highlights gaps in our knowledge on the efficacy of on-farm and factory processing food-chain interventions aimed at reducing rates of contamination on cattle, pigs and particularly broiler chickens, where further research would be helpful. There has been a decrease in *Campylobacter* levels on poultry over the last 5 years in the UK but levels of human campylobacteriosis cases have remained static. It is not yet known whether this is due to *Campylobacter* levels on poultry being a poor measure of risk from consuming poultry meat, an increase in the consumption of chicken, an increase in the number of people over 60 years of age who are more susceptible to *Campylobacter*, or whether the risks from consuming other meats or becoming infected from non-food sources has increased [4,6].

The Advisory Committee on the Microbiological Safety of Food recommends a multi-prong approach to tackling disease from *Campylobacter* combining interventions across the entire food system including non-poultry livestock [1]. Our survey of the evidence supports this recommendation as there is no evidence that any single intervention has a major effect, whereas concerted multiple-intervention campaigns in Iceland and New Zealand have had some effect. Nevertheless, to implement more effectively such a ‘multiple-hurdle' strategy, it would be helpful to conduct more whole food-chain studies that robustly quantify the likely main environmental sources of *Campylobacter* and then go on to analyse the effect of specific on-farm and in-factory interventions on changing the numbers and types of *Campylobacter* on final food products. Experimental and modelling work that evaluates how different interventions interact and combine across the food-chain to reduce levels on retail products would be particularly valuable [7]. The increasing availability of whole-genome DNA sequencing approaches combined with epidemiological and classic microbiological methods offers new tools to understand *Campylobacter* origins, transmission and disease (e.g. [7]). Lastly, we need to better understand the behavioural science of how people assess and understand the risks of food poisoning from *Campylobacter* (and of course other agents), and how they can be empowered to protect themselves and other people.

## Data Availability

The data are provided in electronic supplementary material [8].

## Appendix

**(a) Aims and scope**

1. *Campylobacter* bacteria are a major cause of acute gastroenteritis, affecting around 600 000 people a year in the UK and over 150 million people a year globally. The economic burden of identified *Campylobacter* cases in the UK, in terms of costs to the healthcare system and the patient, is estimated to be £50 million per annum. The total UK societal cost from just foodborne *Campylobacter,* based on 299 000 cases, is estimated at over £700 million per annum.
2. The aim of this Restatement is to summarize succinctly the natural science evidence base concerning the origin and transmission dynamics of *Campylobacter* species causing human disease to assist policy making. The Restatement also summarizes evidence for the efficacy of different interventions intended to control *Campylobacter.* The focus is on evidence of greatest applicability to the UK. It also provides a consensus judgement by the authors on the nature of the different evidence components, and a consensus was arrived at using the studies listed in the annotated bibliography. We use the following descriptions, which explicitly are not a ranking, indicated by abbreviated codes. Statements are considered to be supported by **[S_trong_]**, **[L_imited_]**, **[E_xp_O_p_]** and **[P_roj_]** codes, which are defined in the Materials and methods section. Codes at the end of sections and sub-sections after full-stops indicate they apply to the whole previous section; codes preceding full-stops or within sentences apply to that sentence or clause only. Throughout the restatement, we use the terms ‘infection' and ‘infected' to mean the presence of *Campylobacter* bacteria in animals (humans and farmed animals) regardless of whether they cause disease. World Bank country classifications are used throughout. The abbreviation ‘95% UI' denotes the 95% uncertainty interval.

**(b) *Campylobacter* disease in humans**

3. *Campylobacter* can cause acute diarrhoea and gastroenteritis in humans when ingested. *Campylobacter* may derive from food or non-food sources (such as lakes when swimming). Symptoms of campylobacteriosis range from mild to severe, and usually resolve by themselves in around a week. Frail and immunocompromised individuals have greater susceptibility to campylobacteriosis **[S_trong_]**. Only a few studies exist that monitor the effects of deliberate exposure of humans to *Campylobacter*, for understandable ethical reasons, and one shows illness can be caused by the ingestion of as little as a few hundred bacterial cells, but not all humans infected with *Campylobacter* showed signs of disease **[L_imited_]**.
4. The species *Campylobacter jejuni* and *Campylobacter coli* are the major causes of campylobacteriosis globally. In the UK, *C. jejuni* is responsible for approximately 10 times more cases of human disease than *C. coli* **[S_trong_]**.
5. In high income countries, analyses conducted under the assumption that all ages are equally as likely to present to GPs show cases of laboratory confirmed campylobacteriosis are most frequent in children under 5, young adults (aged 20–30), and in those aged over 60 **[S_trong_]**. In low and lower- and upper-middle income countries, illness from *Campylobacter* infection occurs most frequently in children under 2, with severity of symptoms inversely related to age in older individuals **[L_imited_]**.
6. Comparing the incidence of campylobacteriosis across continents and analysing global trends is hard due to differing sampling and reporting conventions, frequent under-reporting (particularly in countries where many uninsured people have to pay for healthcare), and very low surveillance levels in low income and lower- and upper-middle income countries. The WHO estimates 166 million cases of campylobacteriosis occurred worldwide in 2010 (95% UI: 92–301 million) causing 37 600 deaths (95% UI: 27 700–55 100). In 2018, the Centres for Disease Control and Prevention in the USA reported that 20 campylobacteriosis cases were diagnosed for every 100 000 people but that many more cases went undiagnosed or unreported, and they estimated *Campylobacter* infection affects 1.5 million US residents every year. In low income countries, high burdens of *Campylobacter* in children under two are correlated with stunting **[L_imited_]**.
7. There is greater consistency of reporting methods among European countries making these more comparable but difference in access to and costs of healthcare across Europe may skew this. *Campylobacter* was the most commonly reported gastrointestinal bacterial pathogen in humans in the EU in 2019 with over 220 000 confirmed cases, and an average reported incidence of 60 per 100 000 population. European campylobacteriosis rates have remained stable between 2015 and 2019, and are more than twice that of salmonellosis which is caused by the next most prevalent gastrointestinal bacterial pathogen (*Salmonella*). A decrease of 100 000 campylobacteriosis cases was reported in Europe in 2020 (120 000 cases) compared to 2019: it is likely this drop was due to various effects of the COVID-19 pandemic and corresponding social lock-downs making it hard to compare to preceding years. UK campylobacteriosis rates have fluctuated between 86 and 114 reported cases per 100 000 between 2006 and 2017 with no overall trend. In 2019, the UK reported a campylobacteriosis rate of 88 cases per 100 000 population. Rates in the UK have thus consistently exceeded the EU average since 2015. Studies in England and Wales between 1989 and 2011 show campylobacteriosis cases are increasing in those aged over 50 **[S_trong_]**.
  a. For every case recorded by UK national surveillance centres, around 8–9 are estimated to occur in the community without being reported, so calculations of the economic costs of campylobacteriosis based solely on reported cases are an underestimate. Extrapolations from reported data estimated *Campylobacter* to be the largest cause of bacterial food poisoning cases in the UK underlying 299 392 (95% CI 127 128–571 332) or 33% (95% CI 18%-47%) of food-related infections resulting in the greatest number of GP consultations (42 506; 95% CI 18 683–75 857) for foodborne illness in 2018. After correcting for under-reporting, the rate of campylobacteriosis in the England and Wales population is estimated to have remained roughly stable at approximately 1000 cases per 100 000 since 2009 **[P_roj_]**.
  b. Modelling of clinical data indicate *Campylobacter* caused the greatest number of hospitalizations of foodborne diseases in 2018, and approximately 1% of UK all foodborne campylobacteriosis cases led to hospitalization (median 3505 per year, 95% CI 1352–7641). Estimates of deaths due to campylobacteriosis are around 45 people per year in the UK (95% UI: 24–84) **[P_roj_]**, and foodborne *Campylobacter* was involved in 21 UK deaths in 2019 **[P_roj_]**. For comparison, influenza was involved in 1213 deaths in England and Wales in 2019 **[S_trong_]**. Estimating rates of death due to *Campylobacter* is difficult because the impact of campylobacteriosis may be masked or exacerbated by other health problems or by not being properly recorded **[E_xp_O_p_]**.
  c. Modelling analysis indicates that 70 000 quality-adjusted life years (QALYs) are lost per year in the UK due to just foodborne *Campylobacter,* but with considerable uncertainty (95% UI: 40 000–108 000). Norovirus imposes the largest burden among foodborne diseases and is responsible for 3.5 times more lost QALYs than *Campylobacter*, which imposes the next greatest burden. For comparison, influenza is estimated to be responsible for a loss of around 30 000 QALYs with standard vaccination rates **[P_roj_]**.
8. The majority (99%) of campylobacteriosis cases represent isolated infections of individuals, although single-source outbreaks can occur. The number of reported outbreaks in the UK ranged from 5 to 22 per year between 2006 and 2016, with the numbers of people affected per outbreak between 2 and 167. The largest recorded outbreak of campylobacteriosis globally occurred in Havelock North, New Zealand in 2016 when over 5000 people were infected by *C. jejuni* from contaminated untreated drinking-water boreholes **[S_trong_]**.
9. Campylobacteriosis incidence in some high income countries shows marked seasonality. In the UK, the total number of campylobacteriosis cases is greatest in early summer, peaking during May and June **[S_trong_]**. There is some evidence that the seasonal variation is more marked in rural than in urban areas and in infants under five **[L_imited_]**. No factor has been proven to drive these seasonal patterns although a number of hypotheses, for example higher temperatures, increased barbeques, prevalence of flies attracted to food, have been suggested **[E_xp_O_p_]**.
10. A fraction of campylobacteriosis cases leads to longer-term health conditions such as Guillain–Barré syndrome (between 1 per 1000 and 1 per 5000 cases), reactive arthritis (9 per 1000 cases) and post-infectious irritable bowel syndrome (up to 33 per 1000 cases) **[S_trong_]**. The role of *Campylobacter* in the development of these clinical conditions is imperfectly understood and these conditions may also be caused by other infections.
11. **Summary:** *Campylobacter* is the major cause of bacterial gastroenteritis in the UK and around the world. *Campylobacter* infections usually cause short illness but because *Campylobacter* prevalence is relatively high this translates into a significant socioeconomic burden. Infrequently, *Campylobacter* infections lead to more serious outcomes, including death, with the young, elderly and infirm at greatest risk. In low income and lower- and upper-middle income countries, *Campylobacter* is endemic and a major cause of childhood diarrhoeal illness.

**(c) How humans become infected with *Campylobacter***

12. *Campylobacter jejuni* and *C.* *coli* are bacteria commonly found in the intestines of domesticated and wild animals, especially birds. *Campylobacter jejuni* tends to be the dominant species in cattle, sheep, broiler chickens and turkeys, and *C. coli* tends to be the dominant species in pigs **[S_trong_]**.
13. *Campylobacter* has also been isolated from several different environmental sources, including soil, water and sewage. The most likely explanation for the presence of *Campylobacter* in water and soils is shedding by animals **[E_xp_O_p_]**. Survival of *Campylobacter* outside the gut is poor relative to many other species of pathogenic bacteria, with the bacteria demonstrating relatively high sensitivity to oxygen, drying, freezing and low pH **[S_trong_]**.
14. *Campylobacter* can persist outside of animal guts in the environment for short periods; for instance, for several weeks in groundwater **[S_trong_]**. Animal guts have stable temperatures and very low levels of oxygen and so it is of note that some strains display greater tolerance to elevated oxygen levels or extremes in temperature **[S_trong_]**, which may aid survival outside of animal guts **[E_xp_O_p_]**. Persistence in the environment has also sometimes been associated with the presence of certain protozoa (some bacteria persist in the environment in the bodies of other organisms) **[L_imited_]**. Some but not all *Campylobacter* variants may form their own biofilms (a community of cells adhering to each other and to a surface). It is possible that survival in the environment may be enhanced by attachment to existing biofilms of other species **[E_xp_O_p_]**.
15. Under laboratory exposure to adverse environmental conditions such as prolonged immersion in water or successive freezing-and-thawing, some *C. jejuni* may form viable but non-culturable cells (VBNC) which have low metabolic activity, do not divide, and cannot be resuscitated by conventional culturing techniques. The conditions that promote recovery from a VBNC state, and whether VBNC *Campylobacter* can cause campylobacteriosis, are not understood, and so the biological and epidemiological importance of VBNC *Campylobacter* is uncertain **[L_imited_]**.
16. *Campylobacter* species are genetically diverse, with new genotypes continually being identified. Genetic variation among *Campylobacter* is not continuous but tends to cluster into various distinct groups (clonal complexes) which can be identified using DNA sequencing. These clonal complexes appear stable over time and space, and some, but not all, are tightly associated with particular types of host animal **[S_trong_]**.
  a. Several *Campylobacter* clonal complexes are predominantly (but not exclusively) associated with a particular host species, such as ST-257 (ST stands for sequence-type) in chickens or ST-61 in cows, but some, known as ‘multi-host complexes' or ‘generalist lineages', such as the ST-21 complex, are found across multiple animal host species **[S_trong_]**. There is a suggestion that the intensification of beef production may have provided opportunities for the specialization of some *C. jejuni* sequence types **[L_imited_]**.
  b. In general, wild bird species have their own *Campylobacter* types, and these are distinct from those found in domestic birds such as chickens and farmed ducks; however, some multi-host sequence-types are found in both poultry and wild birds **[S_trong_]**.
17. Genetic attribution studies use knowledge of *Campylobacter* sequence-types that are closely associated with particular types of animals to identify the likely source of *Campylobacter* isolated from human disease cases (see box 1 in the annotated appendix). In the UK, as in other high income countries, host-associated genotyping using genetic markers has shown that the large majority (greater than 95%) of campylobacteriosis cases match *Campylobacter* genotypes that are associated with agricultural livestock. Recent attribution studies in the Netherlands and France using whole genome sequences from hundreds of isolates concluded the main sources of campylobacteriosis were from livestock (78% of cases) but that non-food sources (such as pets and water) were also a significant cause of campylobacteriosis (22% of cases). Two in three livestock cases derived from poultry and the rest from ruminants in studies from France and the Netherlands. Attribution studies using *C. jejuni* isolated from human disease cases in the UK show these predominantly involve genotypes associated with commercial poultry (average 47%, range 19–68%) and then sheep and cattle (average 38%, range 28–54%). *Campylobacter coli* causes one-tenth of human campylobacteriosis cases, but genetic attribution studies indicate these are more likely to derive from ruminants (54%), than poultry (40%) or pigs (6%) **[S_trong_]**.
  a. Some chicken-related clonal complexes (such as ST-661) appear relatively abundant but cause disproportionately fewer cases of human disease than would be predicted given their prevalence. Other types (ST-21) have been reported to increase in relative abundance from farm to clinical cases **[L_imited_]**. It is not yet clear if different *Campylobacter* genotypes differ in their ability to cause human disease **[E_xp_O_p_]**.
18. Genetic attribution studies have shown that non-agricultural animal-associated *Campylobacter* types, for example types found in wild birds, can cause human disease but at substantially lower levels (under 5% of cases) than those associated with livestock **[S_trong_]**.
19. Epidemiological studies correlating disease incidence with risk factors in Europe, Australasia and the USA show both sporadic infections and outbreaks of campylobacteriosis are correlated with the consumption of poultry products such as chicken meat and chicken liver (see box 1 in the annotated appendix). Other risk factors that have frequently been identified include: contact with poultry; handling and eating raw or undercooked meat and seafood; consumption of raw milk; contact with farm animals; contact with companion animals, especially dogs; exposure to environmental water bodies (e.g. lakes); and the consumption of untreated water. The reservoir of *Campylobacter* in poultry is estimated to be responsible for between 50 and 80% of human campylobacteriosis cases **[S_trong_]**.
20. International travel has also been identified as a risk factor for campylobacteriosis **[S_trong_]**. One study suggested that 17–18% of recorded cases in the UK are associated with travel outside the country of residence **[L_imited_]**.
21. Risk factors identified in epidemiological studies can change in importance over time and new risk factors can emerge, for instance, contact with garden soil has only recently been identified as a risk factor. There is an increasing incidence of sporadic cases of campylobacteriosis related to the consumption of unpasteurized milk in the UK and USA **[S_trong_]**.
22. Epidemiological and genetic analyses of campylobacteriosis outbreaks demonstrate these mostly derive from single point sources that directly contaminate many people; human to human transmission is rare (around 3%) in outbreaks. The major risk factors associated with outbreaks are contaminated drinking water and the consumption of raw milk and chicken-liver pâté **[S_trong_]**.
23. Some outbreaks of campylobacteriosis are diffuse, having a common source but not necessarily clustered geographically **[S_trong_]**.
24. In surveys of fresh UK-produced whole chicken at retail outlets by the UK's Food Standards Agency (FSA), the proportion that tested positive for *Campylobacter* using standard microbiological methods dropped from 73 to 40% between 2014 and 2018 **[S_trong_]**. A recent Scottish study reported an incidence of 0.1% (95% UI: 0–0.7%) on retail fresh beef mince sampled in 2019. There are no comparable recent surveys for other food products in the UK. Limited sampling of beef, pork and sheep between 2003 and 2007 in the UK found mean *Campylobacter* prevalence in the range of 0.3–16% **[L_imited_]**.
25. *Campylobacter* prevalence derived from surveys of UK poultry in shops in 2017 and 2018 by the FSA (see paragraph 24) allows the prediction that exposure to as little as 10 g of even the lowest contaminated UK retail poultry samples may be sufficient to cause campylobacteriosis, if food is not cooked sufficiently or if food preparation hygiene is poor, as this represents a few hundred *Campylobacter* cells (see paragraph 3) **[L_imited_]**.
26. Retail surveys in the USA indicate that around 20% of poultry breast meat was positive for *Campylobacter* in 2018, but differences in sampling methods mean this cannot be meaningfully compared the UK retail survey data.
27. **Summary:** attribution studies, together with risk exposure information based on food surveys, consistently identify meat products as substantial risks for campylobacteriosis. The majority of human *Campylobacter* infections are *C. jejuni* and result from contact with livestock or consumption of meat, with poultry being the most important source followed by ruminant meat. Cases of campylobacteriosis in the UK remain constant despite a decreasing prevalence of *Campylobacter* on poultry in retail outlets.

**(d) How *Campylobacter* is transmitted between animals in agriculture**

**Commercial poultry**

28. In domesticated poultry, *Campylobacter* is commonly considered a commensal (i.e. it causes no harm to the host), but in some circumstances, it may act as an opportunistic pathogen **[L_imited_]**.
  a. In commercial rearing facilities, most infections with *Campylobacter* result in no obvious signs of disease in chickens. Statistically significant relationships between *Campylobacter* infection of broiler flocks and broiler health and welfare markers such as the presence of digital dermatitis and body weight have been recorded in a limited number of commercial operations in the UK **[L_imited_]**. The direction of causality these limited studies is unclear: birds with poor health and welfare may be more susceptible to *Campylobacter* infections, but generally, flocks with both poor and good welfare are infected with *Campylobacter* **[E_xp_O_p_]**.
  b. Stress and immunosuppression in chickens may increase the capacity of *Campylobacter* to move beyond the gut and invade tissues such as muscle and liver **[L_imited_]**.
29. Once introduced into a population of chickens in a broiler unit, *Campylobacter* spreads rapidly via the faecal–oral route and virtually all animals become infected within a week **[S_trong_]**.
30. Broiler poultry do not have contact with their parents after hatching. Vertical transmission from breeder to broiler chickens via eggs both internally and from external contamination has been excluded as a major transmission route because live *Campylobacter* has not been detected in eggs or in chicks under 1 week old. However, the increasing sensitivity of sampling and detection methods, including genetic approaches, suggests vertical transmission may occur rarely (at less than 1 in 60 000 chicks) **[L_imited_]**.
31. Epidemiological investigations have attempted to identify risk factors correlated with the infection of flocks in poultry units. This is difficult due to problems with identifying causality, correlation of risk factors, and considerable farm to farm variability **[E_xp_O_p_].** The most implicated risk factors are:
  a. Poor biosecurity (procedures designed to prevent the introduction and spread of disease-causing organisms), including close proximity of other animals (livestock, pets and rodents), partial depopulation (thinning), and poor poultry welfare correlate with *Campylobacter* infections. However, the presence of *Campylobacter* *per se* is not an indicator of poor welfare **[S_trong_]**.
  b. There is some evidence that the use of untreated drinking water for poultry is a risk factor **[L_imited_]**.
  c. Flies and other insects are able to vector *Campylobacter* **[S_trong_]**. The presence of insects is identified as a risk factor in some, but not all, surveys **[L_imited_]**.
32. *Campylobacter* can be found in the environment around poultry houses, both before and after a cohort of birds is introduced. The genetic types of *Campylobacter* isolated from the environment surrounding houses are often identical to those found in infected flocks. Similarly, *Campylobacter* types in feed distribution and storage systems, litter, transport crates and external or internal drinking water sources are often the same as those in infected chickens **[S_trong_]**. The epidemiological interpretation of these observations is difficult as the direction of transfer (flock to environment or environment to flock) is typically not known **[E_xp_O_p_]**.
33. Several studies have shown that inadequate cleaning and disinfection between successive flocks in a poultry house is correlated with subsequent *Campylobacter* infection. These studies are unable to show directly if previous flocks or the house/farm generally were the original source of infection **[L_imited_]**.
34. There is evidence that employees entering and moving between different poultry sheds (for example to remove particular birds) is correlated with higher levels of *Campylobacter* infection **[S_trong_]**.
35. The preparation and transport of flocks to slaughterhouses increases the risk of *Campylobacter* transmission from catching crews, and between farms via transportation equipment such as crates, in some but not all studies **[L_imited_]**.
36. Organic and free-range systems where birds have outdoor access are at a greater risk of *Campylobacter* infection than intensively reared flocks (permanently in sheds), and molecular typing studies suggest contamination was from wild birds and other livestock **[S_trong_]**. However, there is no clear evidence as to whether this translates to differences in contamination levels on retail poultry products **[L_imited_]**. Slower growing free-range or organic flocks are usually slaughtered when 63–81 days old versus 35–42 days for conventionally reared flocks. Age of birds at slaughter in short-lived broilers is a frequently identified risk factor for flock contamination **[S_trong_]**, presumably because living longer increases the chances of infection.

**Commercial pigs and cattle**

37. Evidence for the origin and transmission of *Campylobacter* in pigs and cattle is limited.
38. Pigs are infected with *Campylobacter*, especially *C. coli*, from other herd members less than a week after birth, and prevalence increases through the production cycle from 0% at birth, 33 to 48% 1-week post-birth and 67–96% in finishing pigs around six months old **[S_trong_]**.
39. Pigs are typically asymptomatic *Campylobacter* carriers but there is some evidence of an association of infection with post-weaning diarrhoea and lower back-fat and weight gain **[L_imited_]**.
40. *Campylobacter*, especially *C. jejuni,* are found in healthy ruminants, and are easily passed between herd members via the faecal–oral route. The *Campylobacter* prevalence in sheep and cattle in the UK is poorly characterized **[L_imited_]**.
41. *Campylobacter* infection in ruminants is associated with higher incidence of abortions **[S_trong_]**.
42. **Summary:** *Campylobacter* is found in a range of livestock species and their associated habitats. Pigs and ruminants are likely infected with bacteria from herd members. Broiler poultry chicks are unlikely infected by bacteria from their parents and there is very limited evidence to understand the sources of poultry infection. In broiler poultry, poor flock health and poor house biosecurity are correlated with increased *Campylobacter* infection making it hard to disentangle the cause of infections. Records of *Campylobacter* prevalence in livestock other than poultry are poor.

**(e) Food chain interventions to control levels of *Campylobacter* on retail produce**

**On-farm interventions**

43. In a typical broiler chicken operation, some birds are harvested at around 35 days (partial depopulation) and then the majority of birds harvested at 42 days. In a large field trial in the UK, flocks with enhanced biosecurity interventions were 25 and 50% less likely to be infected with *Campylobacter* at partial and final depopulation, respectively **[S_trong_]**.
  a. Chlorination and/or the acidification of drinking water may reduce *Campylobacter* levels in poultry digestive tracts on farms, but the effect is inconsistent across studies **[L_imited_]**. No consistently positive effects have been found on *Campylobacter* levels from the provision of chicken feed additives, including microbial probiotics, organic and fatty acids, and essential oils. How *Campylobacter* behaves in the gut and its interaction with the rest of the gut microbiome is not well understood **[L_imited_]**.
44. No commercial vaccine currently exists for the prevention of enteric *Campylobacter* infection in animals. There is not a good understanding of the antigens that confer immunity to *Campylobacter* in chickens but these appear strain-specific **[L_imited_]** which makes the production of a general vaccine challenging though this is an area of active research.
45. There are no studies that have systematically evaluated the effect of interventions relating to transport and holding practices of livestock, including poultry, on the public health risk of *Campylobacter* **[E_xp_O_p_]**.
46. There are no studies that have systematically evaluated the effect of on-farm interventions on *Campylobacter* prevalence in ruminants or pigs **[E_xp_O_p_]**.

**Processing interventions**

47. *Campylobacter* resides in the intestines of live animals and can be spread to carcasses during post-slaughter evisceration. The majority of available data on the effects of processing on *Campylobacter* contamination are derived from poultry, and there is little information for other livestock species.
48. A number of analyses show a positive flock-level association between the prevalence of *Campylobacter* in broiler chicken intestines (particularly caeca) and the frequency of *Campylobacter*-contaminated carcasses post-slaughter **[S_trong_]**.
49. For poultry, carcass contamination is primarily found on the neck skin as evisceration occurs through the neck and carcasses are subsequently hung upside down. *Campylobacter* is found at the same rates in chicken livers as on neck skins. Where *Campylobacter* is present in a flock, contamination may be found in breast meat in around 5–10% of *Campylobacter* positive carcasses. There is less evidence about the distribution of *Campylobacter* prevalence on other animal carcasses, but meat from larger ruminants is usually sold pre-portioned or processed and infrequently includes skin tissue **[L_imited_]**.
50. The nature of poultry carcass processing procedures can affect the extent of *Campylobacter* spread over carcasses. For example, there is some evidence that visceral rupture can increase *Campylobacter* contamination across the whole carcasses by up to 10-fold **[L_imited_]**. Attention to processing details, such as ensuring the correct settings on machines for the size of the bird, has the potential to reduce the spread of contamination **[E_xp_O_p_]**.
51. Methods where the carcass outer surface is frozen without freezing muscle may reduce *Campylobacter* levels with reductions by forced air chilling of one-half and crust freezing up to 30-fold **[L_imited_]**.
52. Heat treatment of poultry carcasses by steam or water-dipping can reduce *Campylobacter* loads by 10- to 100-fold; but hot water baths may serve as a reservoir for contamination if the temperature is not maintained or hygiene is otherwise poor. Combined steam and ultrasound treatments can reduce *Campylobacter* carcass loads by 300-fold **[L_imited_]**. Heat treatments need to be very carefully monitored to avoid part-cooking **[E_xp_O_p_]**.
53. The application of chlorine and other chemical rinses such as peracetic acid can achieve 10- to 100-fold reductions in levels of *Campylobacter* on poultry carcasses **[S_trong_]**. However, there is no clear evidence that extensive and long-term use of chlorine rinses in other countries (such as the USA) has resulted in lower levels of *Campylobacter* prevalence on raw poultry or rates of campylobacteriosis compared to European countries where the use of chlorine is banned **[P_roj_]**. Concerns have been raised about the introduction of chemicals to the food-chain, and that the use of rinses may lead to a false sense of security and the relaxation of biosecurity on farms **[E_xp_O_p_]**.
54. Irradiation and UV-light exposure can reduce poultry *Campylobacter* loads by 5- to 10-fold **[S_trong_]**. These treatments are demanding of space, time and energy and require workers to be protected from accidental exposure **[E_xp_O_p_]**.
55. Processing interventions focusing on the external surface of carcasses (chemical rinses, crust freezing or chilling, heat treatment and irradiation) address risks associated with carcass surface contamination, but not internal contamination such as of viscera. Such treatments may also affect customers' perception of the freshness of the product and hence sales **[E_xp_O_p_]**.
56. Freezing the entire carcass can reduce *Campylobacter* levels on poultry by approximately 30-fold and the freezing of livers can lead to a 100-fold drop **[L_imited_]**. Implementation of a carcass freezing policy together with a national surveillance programme was considered a critical part of the control of epidemic campylobacteriosis in Iceland **[E_xp_O_p_]**.
57. Levels of *Campylobacter* on chicken products decrease after packing and during chilled shelf life **[S_trong_]**.
  a. Experimental studies show packaging chicken under controlled atmospheres (particularly high levels of O_2_ with mixes of N_2_ and CO_2_), especially in conjunction with reduced temperatures, can reduce *Campylobacter* loads by 100- to 10 000-fold **[L_imited_]**.
  b. Roast-in-the bag packaging was introduced in the UK to reduce the risk of cross-contamination in the household. Packing interventions were part of the combination of interventions which contributed to the reduction of epidemic disease in Iceland **[P_roj_]**.
58. Some evidence shows *Campylobacter* may be reduced to undetectable levels on the surfaces of pork carcasses with the use of blast-chilling **[L_imited_]**. Specific interventions to control *Campylobacter* on beef are not implemented due to the assumption that measures targeted at other microbiological hazards will also control *Campylobacter*, though this has not been tested **[E_xp_O_p_]**.
59. Milk pasteurization is effective at controlling *Campylobacter,* which can be inferred by the association of campylobacteriosis only with the consumption of unpasteurized raw milk (or when pasteurization fails) **[P_roj_]**.

**Interventions aimed at consumers**

60. Surveys indicate that consumers believe risks in domestic environments are small and awareness of *Campylobacter* risks is low relative to other foodborne diseases **[L_imited_]**; however, the majority of sporadic cases of campylobacteriosis are associated with food prepared and consumed at home **[L_imited_]**. Cross-contamination from fresh chicken meat to other foods via hands and food preparation equipment is the main route of human exposure **[L_imited_]**. Washing raw chicken, a common practice among older consumers, is thought to be a risk factor for cross-contamination **[E_xp_O_p_]**. The consumption of raw milk and undercooked chicken livers is also a known risk factor for illness and is implicated in outbreaks **[S_trong_]** but it is not clear the degree to which this is understood by the general public in the UK **[E_xp_O_p_]**.
61. Research into human vaccines, particularly for overseas travellers and the military, is underway. Several candidates have been tested on humans but none have to date conferred sufficient protection **[L_imited_]**.

**Coordinated interventions**

62. Coordinated national campaigns in the UK, Iceland and New Zealand, implementing a range of voluntary and regulatory interventions across the production chain from farm to consumer education, have resulted in reductions in rates of *Campylobacter* on poultry and/or campylobacteriosis. The precise drivers for decreases are not easy to disentangle as many changes were applied together.
63. Following a spike in campylobacteriosis in the late 1990s, peaking at 118.2 laboratory reported incidences per 100 000 in 1999, Iceland implemented a series of control measures including enhanced surveillance, increased biosecurity, changes in poultry processing and consumer education. Rates of campylobacteriosis fell to an average of 20.5 incidences per 100 000 in the period 2002–2007 **[S_trong_]**. While it is not possible precisely to identify the most effective intervention, freezing meat from *Campylobacter*-positive flocks prior to sale was thought to be the most important **[E_xp_O_p_]**.
64. Before 2006, New Zealand had high rates of campylobacteriosis compared with other high income countries, peaking at 396 reported cases per 100 000 population in 2003. In 2006, a range of voluntary and regulatory interventions targeted at all levels from the farm to consumer education were implemented and levels of campylobacteriosis dropped 54% by 2008 **[S_trong_]**. Monitoring and reporting of *Campylobacter* levels on chicken carcasses, and the setting of mandatory performance targets, were considered to be the most important interventions **[E_xp_O_p_]**.
65. **Summary**: On-farm enhanced biosecurity interventions can correlate with lower levels of *Campylobacter* on poultry*.* There is no single poultry processing intervention that provides perfect control of *Campylobacter*, but a combination of the use of cold or heat carcass treatments, and better packaging may reduce overall *Campylobacter* levels on retail poultry, although the evidence base on the efficacy of particular treatments is limited. There is a lack of evidence for how multiple on-farm and processing interventions may interact in terms of *Campylobacter* control. There is a lack of evidence for the value of on-farm and processing interventions for non-poultry livestock.

**(f) Antimicrobial resistance**

66. *Campylobacter* can acquire antimicrobial resistance through a number of mechanisms, including novel mutations and the acquisition of resistance genes via horizontal gene transfer from other bacteria **[S_trong_]**.
67. Antimicrobial drugs are used in poultry production to prevent and treat a wide range of bacterial diseases. Poultry are not treated for *Campylobacter* directly, but *Campylobacter* in the poultry caeca and gut will be exposed to any antimicrobials administered. Poultry medications, including antimicrobials, are usually given at the flock level via feed or drinking water **[S_trong_]**.
68. The most commonly used antimicrobials to treat disease in UK poultry production are fluoroquinolones, followed by penicillin. As a response to concerns over antimicrobial resistance, and a halt in the use of antibiotics as growth promoters in 2006, overall antimicrobial use in the UK poultry industry declined 80% from 2013 to 2017 **[S_trong_]**.
69. Globally, antimicrobial resistance in *C. jejuni* and *C. coli* has increased in recent years in both human and animal isolates, with high levels of resistance to fluoroquinolones and macrolides and emerging resistance to aminoglycosides. There is increasing evidence for resistance to other antimicrobials and the emergence of multi-drug resistant strains **[S_trong_]**.
70. Over time, antimicrobial use in the poultry industry is correlated with antimicrobial-resistant *Campylobacter* in humans. There has been a steep and sustained rise in the incidence of disease caused by fluoroquinolone-resistant *Campylobacter* in the UK from 1997 onwards **[S_trong_]**. The proportion of *Campylobacter* resistant to antimicrobials is greater on farms which use antimicrobials than those which do not **[S_trong_]**.
71. *Campylobacter* readily acquires resistance to fluoroquinolones via simple genetic mutations, and resistance is not lost even if fluoroquinolones are withdrawn as resistant types have an advantage over non-resistance types **[S_trong_]**.
72. Resistant infections are disproportionally associated with international travel **[S_trong_]**, presumably to areas where antibiotics are more commonly used in livestock and humans **[E_xp_O_p_]**.
73. Routine antimicrobial treatment of individuals with campylobacteriosis is not usually recommended, as antibiotics only shorten the duration of disease by an average of 1.3 days **[S_trong_]**. Antimicrobials are recommended in severe cases, typically immunocompromised patients or young children.
  a. People infected with resistant *Campylobacter* may experience illness that is prolonged and more severe than those infected with sensitive strains **[L_imited_]**. It is not clear whether resistant strains tend to possess additional virulence factors.
  b. Fluoroquinolone-resistant *Campylobacter* is designated by the WHO as a ‘high priority' pathogen for new antibiotic research and development.
  c. In the USA, one quarter (23%) of *Campylobacter* isolates were reported to be resistant to fluoroquinolones, and these were associated with approximately half of the 65 deaths per year involving *Campylobacter* in 2013.
74. There is no current evidence that antimicrobial-resistant strains of *Campylobacter* behave differently in the food chain relative to non-resistant strains in terms of their sensitivity to control interventions **[E_xp_O_p_]**.
75. **Summary:** the use of fluoroquinolones in poultry production has been consistently associated with the level of resistance in *Campylobacter* isolates from poultry and human cases, and the prevalence of fluoroquinolone resistance in poultry and human derived isolates are both increasing despite the reduction in use of antibiotics for livestock in the UK.
